# Time and Motion Study of a Community Patient Navigator

**DOI:** 10.3934/publichealth.2014.2.51

**Published:** 2014-04-06

**Authors:** Sara S. Phillips, Laura S. Tom, Charito Bularzik, Melissa A. Simon

**Affiliations:** 1 Obstetrics and Gynecology, Northwestern University Feinberg School of Medicine, Chicago, IL, 60611, USA; 2 Rush Medical College, Chicago, IL, 60612, USA; 3 Access DuPage, Carol Stream, IL, 60188, USA; 4 Department of Preventive Medicine, Northwestern University Feinberg School of Medicine, Chicago, IL, 60611, USA; 5 Department of Medical Social Sciences, Northwestern University Feinberg School of Medicine, Chicago, IL, 60611, USA

**Keywords:** patient navigation, time in motion analysis, breast cancer, uninsured, health services

## Abstract

Research on patient navigation has focused on validating the utility of navigators by defining their roles and analyzing their effects on patient outcomes, patient satisfaction, and cost effectiveness. Patient navigators are increasingly used outside the research context, and their roles without research responsibilities may look very different. This pilot study captured the activities of a community patient navigator for uninsured women with a positive screening test for breast cancer, using a time and motion approach over a period of three days. We followed the actions of this navigator minute by minute to assess the relative ratios of actions performed and to identify areas for time efficiency improvement to increase direct time with patients. This novel approach depicts the duties of a community patient navigator no longer fettered by navigation logs, research team meetings, surveys, and the consent process. We found that the community patient navigator was able to spend more time with patients in the clinical context relative to performing paperwork or logging communication with patients as a result of her lack of research responsibilities. By illuminating how community patient navigation functions as separate from the research setting, our results will inform future hiring and training of community patient navigators, system design and operations for improving the efficiency and efficacy of navigators, and our understanding of what community patient navigators do in the absence of research responsibilities.

## 1. Introduction

Over the past decade, patient navigation researchers have validated the efficacy of navigation through studies on timeliness of care, patient satisfaction, patient feedback on barriers to care, patient quality of life, treatment outcomes, and observations on what navigators do [1]–[7]. Patient navigators—individuals trained to provide support and guidance to patients to remove barriers to care—are now recognized as integral intermediaries between medical care teams and patients in both institutional and community settings, enabling patients to more effectively navigate the healthcare system and receive coordinated care. As such, navigators are utilized increasingly in both urban and rural contexts [6]. To date, only two papers used the terms community navigator or community-based navigator. These papers establish how a community navigator works in a non-institutional, community setting serving patients affiliated with many different health institutions. Furthermore, community navigation programs were developed utilizing a community-based participatory research approach and outreach was community-based, not clinic-based [8],[9]. However, the roles and activities of community patient navigators, and those without research responsibilities in particular, remain largely unexplored. This paper will address the latter by shadowing and documenting for three days the activities of a community patient navigator—a patient navigator working in a non-institutional, community setting—who is not engaged in research activities. We use a time and motion approach, which places emphasis on the allocation of time associated with each activity, thereby relying upon precise data collection to inform a more efficient patient navigation process. As with any service that seeks to provide patient support, the time spent on various navigation activities is often unpredictable; patient navigation is a role that requires flexibility. However, gaining insight into how navigators spend their time is crucial not as a predictive tool, but rather as a reflection of the current amount and type of work. This snapshot depicts how the skills of community patient navigators are utilized—in what settings and with what people.

Previous studies assessed the role of navigators through meta-analyses, highlighting based on qualitative impressions the broad range and reactive nature of activities performed by navigators [1],[10]. However, the results of these studies needed further verification by time-stamping the activities of navigators. Other studies filled this gap by accounting for the time intervals associated with each task: examining what navigators do while actively involved in data collection for research purposes [7] or developing protocols for these purposes [11]. Nonetheless, these approaches usually involved time spent by navigators on research data collection, an activity that may be less pertinent outside the research context. Patient navigation in research settings has been depicted as a resource-intensive endeavor [7], thus warranting further exploration of the efficiency of navigators. Our study witnessed community patient navigation for uninsured women with a positive screening test for breast cancer, separate from the research context.

The goal of this pilot study was to ascertain how a community patient navigator free from research duties spent her time. Using time and motion methodology, we performed a detailed analysis of time spent on each activity in order to create a snapshot of the workload of a community patient navigator. As the profession of community patient navigation continues to flourish, such descriptions can guide the work of those interested in health care system design and operations—from human resource managers to researchers and policy makers.

## 2. Materials and Method

This study was conducted as part of the DuPage Patient Navigation Collaborative (DPNC), a five-year community-based participatory research study for uninsured suburban women with a positive screening test for breast or cervical cancer; its primary aim was to reduce the time between abnormal screening and follow-up. The DPNC was a partnership between Northwestern University and Access DuPage, a non-profit organization that facilitates primary care services for over 14,000 residents of DuPage County. A county just west of Chicago, DuPage has a large, primarily uninsured, Latino population. Following completion of the primary DPNC navigation research study, a time in motion study was conducted to document the day-to-day activities of a DPNC community patient navigator no longer involved in research activities.

The female patient navigator was a bilingual English and Spanish speaker, originally from Bolivia, who navigated Spanish-speaking women without English fluency that on screening received abnormal breast or cervical results. Her primary focus was on patients with abnormal breast results, and thus all the patients witnessed during this study fell into this category. She was a lay navigator with a degree in Elementary Education from Bolivia, experience working as a case manager and health educator for the Why Wait Program at the DuPage County Health Department, and a recipient of patient navigation training through Access DuPage. In her 5 years as a navigator for the DPNC, she worked in the Access DuPage office in a windowless room shared with another patient navigator. This room was adjacent to the communal space, and the connecting door—which nearly always remained opened—allowed colleagues easy access to the navigator. Upon the conclusion of the navigation research study, she was no longer required to maintain research logs, participate in other research activities, or communicate with the research team.

### 2.1 Data Collection

We shadowed the patient navigator for three days in May of 2013. Days were chosen by the navigator in order to best represent her typical workload. One of the authors (SP) maintained a written record of the actions performed by the community patient navigator by the minute with a corresponding descriptor of pertinent details, including the people engaged, tasks performed, locale, and rationale for the use of time. This written record was more acceptable and inconspicuous than using an electronic device such as a tablet, which was particularly important for shadowing patient and doctor interactions. These written records were subsequently transferred to an Excel spreadsheet following the visit. This level of detail linked to each minute was grounded on time and motion methodology. Although time and motion conducted via continuous observation is similar to a work sampling method, the detail collected has been shown to be superior in continuous observation [12]. An earlier patient navigator research study conducted by Clark et al continuously observed navigators and recorded observation sessions in 15 minute intervals [7]. We believe our minute-by-minute assessment will yield even greater detail and precision. We opted to use third-party observational records of navigator activities instead of navigator self-report, as observations may be more efficient and yield greater receptivity of the observant [13]. Although there were concerns that individuals may modify their behaviors upon being observed, past observational studies did not witness this Hawthorne Effect [12],[13]. We obtained the patient's permission prior to shadowing the navigator during a patient appointment.

### 2.2 Analysis

The coding of the data into master themes and subthemes was performed by one of the investigators (SP) and then reviewed by a coauthor (LT) for accuracy. Navigator actions were analyzed under three separate, main thematic lenses: *Task*, *Location*, and *People*. The *Task* lens describes the general nature of the activity (e.g., accompanying patients to an appointment), whereas the *Location* lens describes the location where the activity was performed (e.g., hospital), and the *People* lens reflects who was involved in the activity (e.g., patient).

**Table 1.** Time allocation over 3 days by lens: task, location, and people.

*Subtheme*		*Total Minutes*	*Minutes as % of Total*
***Task***			
	Computer/Email	334	25.4
	Accompany Patient To Appointment	335	25.5
	Self	281	21.4
	Inner Office Communication	173	13.2
	Phone Calls	93	7.1
	Transportation	75	5.7
	Logging Navigation Communication	21	1.6
	Paperwork	3	0.2
***Location***			
	Office	825	62.7
	Hospital	335	25.5
	Out	80	6.1
	Car	75	5.7
***People***			
	Individual (Work)	521	39.6
	Patient	318	24.2
	Individual (Personal)	281	21.4
	Team Communication	173	13.2
	Provider's Office Contact	22	1.7

Under each lens, actions were coded into subthemes as represented in Table 1. The activities captured in each subtheme are summarized in the title of the subtheme (e.g., patient contact, team communication, provider's office contact). *Task* subthemes included Computer/Email, Accompanying Patients to Appointment, Self, Inner Office Communication, Phone Calls, Transportation, Logging Navigation Communication, and Paperwork. *Location* subthemes included Office, Hospital, Out and Car. *People* subthemes included Individual (Work), Patient, Individual (Personal), Team Communication, and Provider's Office Contact. Subthemes will be described in further detail in the results, however a few warrant clarification here. The Individual (Work) subtheme under the *People* lens pertained to activities conducted by the navigator without contact with others, whereas the Individual (Personal) subtheme designated time spent on non-work activities such as lunch and short breaks, often with coworkers. Under the *Location* lens, the subtheme Out captured the period of time that the navigator spent waiting for the next appointment after the first was canceled in the same area of town, and the subtheme Hospital includes the time spent waiting at the hospital for the cancelation. In contrast, Car described the time spent traveling between the office and the hospitals in a car.

Time allocated for certain subthemes under the three main thematic lenses reflected the same actions performed and thus display the same number of total minutes under the separate lenses. For example, in quantifying work-related group discussions, Inner Office Communication under the *Task* lens and Team Communication under the *People* lens capture the same number of minutes. One lens describes the task itself and the other lens describes the people involved in the activity. Similarly, the Self subtheme when viewed from the *Task* lens garners the same number of minutes as the Individual (Personal) subtheme when viewed from the *People* lens. By stating that two similarly titled subthemes each viewed under a different thematic lens present with the same number of minutes, we are simply highlighting the consistency of our data.

## 3. Results

### 3.1 Task

Community patient navigator tasks over the three days observed are displayed in Table 1. Notable results emerging from our analysis of tasks include the lack of paperwork and patient logging, which occupied only 0.2% and 1.6% of her time respectively. Over a three day period, paperwork encompassed just one observation of filling out a patient registration form for a patient to be seen later in the week. Although our community patient navigator did not have access to electronic medical records because she was not affiliated with any one health network, she still maintained a log of the patient's medical history as part of her navigation duties. Logging Navigation Communication involved documenting all contact with patients and communication with providers on the patient's behalf in a secure Excel file; this documentation was not required by research and was merely utilized for the organizational purposes of the navigator.

The navigator spent the largest percentage of her time doing computer work (25.4%) and accompanying patients to appointments (25.5%). Her work on the computer included checking and responding to email (e.g., sending an email to a nurse case manager to communicate when a patient was scheduled) and Internet searches. Observed activities in which the navigator accompanied patients to the appointment included time spent with the patient in the waiting room preparing the patient for the interaction with the doctor, interpreting for the patient with the doctor or technician (e.g., during an ultrasound guided breast biopsy), and discussing the appointment with the patient after the clinical encounter.

In contrast to the amount of time spent accompanying patients to appointments, the navigator spent a relatively small percentage of time on the phone (7.1%). But these calls were numerous in number. Navigation tasks observed on the phone included calling the cancer center to request paperwork for a patient who needs a pap smear every year while taking Tamoxifen (the Illinois Breast and Cervical Cancer Screening Program requires justification in order to fund this special case), answering a phone call directed to the general Access DuPage line, calling a new referral who had already scheduled her next appointment on her own but was confused about the “Breast Health Center” which the navigator clarified, trying to reach a patient to fill out a patient registration form, checking in on a patient who underwent surgery the prior week, and conducting a phone call with providers. Some calls led to a cascade of calls—for example, calling a patient to inform her that she needs another mammogram before aspiration, then subsequently calling the hospital to schedule a mammogram and aspiration of a cyst for the patient, and later re-calling that patient to let her know that she scheduled an appointment.

The remainder of the navigator's day involved inner office communication (13.2%), transportation (5.7%) and time spent for herself (21.4%). Inner office communication observed included navigation-related discussion with fellow navigators regarding plans for the day, immigration and the current policies on legality, a patient's status, and patient navigation roles generally. Transportation was simply time spent driving between the office and the hospital to accompany patients to appointments. Time categorized as Self included scheduled lunch breaks, personal calls (e.g., setting up a dentist appointment), and conversations with colleagues during breaks.

### 3.2 People & Location

Our navigator spent the largest portion of her time working independently (39.6%), but nearly a quarter of her time involved direct contact with patients (24.2%). The navigator spent less than 2% of her time (22 minutes) over the three day period in contact with providers' offices outside accompanying patients to their medical appointments. She also spent most of her time in the office (62.7%) or at the hospital (25.5%) and relatively little time driving (5.7%). In an effort to understand how much time she spent driving to appointments relative to the time spent with patients at their appointments, we calculated a ratio correcting for the one appointment that never occurred. The ratio of driving to time spent at appointments was 0.2.

## 4. Discussion

This time and motion study revealed that the community patient navigator spent very little time doing paperwork and patient logging—most likely due to the removal of her research responsibilities. Instead, this community patient navigator spent the majority of her time accompanying patients to appointments, doing computer work and email communications, and communicating with her colleagues. This snapshot portrays the community patient navigator as a versatile, collaborative team player who is highly engaged as a member of the patient's medical care team.

Our study advances the literature by providing an analysis of the roles and responsibilities of a community patient navigator without research activities. Prior studies examining the daily actions of patient navigators assessed patient navigators who were extensively involved in data collection for research purposes. Parker et al. first developed a protocol for determining what patient navigators do, which defined an “Other” category that included “research-related activities” and a “Document/Review” category [11]. Although these categories did not subcategorize hours spent only on research, research activities within the PNRP were a key part of navigators' responsibilities [14]. A subsequent study utilized the protocol to track the activities of 34 navigators across 9 sites, some community-based and others institutional, within the NCI-funded Patient Navigation Research Program (PNRP) [7]. Whereas paperwork and patient logging occupied 0.2% of our community patient navigator's time, documentation and reviewing medical records per research protocol required 48% of navigators' time on average in the PNRP [7]. Moreover, we observed that our navigator spent more time accompanying patients to appointments (25.5%) than PNRP navigators tasked with research responsibilities, where navigators spent only 15% of their time facilitating patient-provider interactions [7],[11].

Interactions with medical providers may be particularly stressful for patients with poor English language skills or limited medical knowledge. Given that our navigator worked with non-English speaking patients, this linguistic support in the context of doctor interactions was particularly important. Therefore, when we consider the ratio of time spent accompanying patients to appointments relative to performing documentation, our community navigator ratio was 112 compared to less than one for the PNRP navigators. Without research activities, navigators have the potential to be much more impactful on the patient's experience in the clinic and can afford to spend less time performing documentation. This could not be assumed prior to our study because navigators' research responsibilities included paperwork and team meetings, and thus the additional time granted by the removal of research responsibilities could hypothetically shift to any activity. Our study demonstrates that navigators prioritized supporting patients in the clinics when research duties were removed.

The low ratio of Transport Time: Time Spent Accompanying Patient to Appointment (0.2) experienced by our community patient navigator is a distinct benefit for both the navigator and patient alike, as navigators maximize their time efficiency by minimizing transport time. This enabled our navigator to spend almost 2/3 of her time in the office and almost 1/3 of her time at the hospital. Although this navigator is part of a community based navigation program that serves patients of DuPage county who seek help from doctors across many clinics and hospitals, this low ratio reflects the central location of Access DuPage relative to the major providers. The location of such community patient navigation offices should be strongly considered when building new navigation programs.

We found that the navigator spent 21.4% of her time on Individual (Personal), non-work, activities. This may reflect the stressful nature of patient navigator work and the need for mechanisms such as regular short breaks and social interactions with peers that facilitate resilience in the face of occupational stress. These breaks included eating lunch and engaging socially with coworkers. Such social interactions may not only strengthen team bonds, but may also provide emotional support to the navigator. A support network is particularly crucial for workers engaged in emotionally draining activities like working with cancer patients, which has been shown to cause occupational stress [15].

Our in-depth descriptions of the activities populating the subthemes under the *Task* lens, underscored the breadth of roles that should be included in a job description for use in the hiring and training of navigators. From a human resources perspective, the type of person hired for any job should be founded upon how the applicant's qualities fit with the required actions of the work. After reviewing the actions of a community patient navigator, we recommend that a navigator possess excellent communication skills, the ability to work in teams, culturally competent and technically proficient medical interpreting skills, and self-organization skills to facilitate orchestration of patient follow-up. As a community-based as opposed to hospital or clinic-based navigator, our navigator did not work with medical records. Therefore, experience working with medical records is not necessary for community patient navigators. Moreover, research skills like survey and interview administration, data collection, and interest in scientific literature are less important for navigators outside the research enterprise.

The limitations of our time and motion pilot study include the fact that we only analyzed one patient navigator across three days. Furthermore, we only observed the navigation of Spanish speaking patients who were receiving follow up for abnormal breast exams. The needs of a Spanish speaking patient might be different than the needs of an English speaking patient, and similarly, the needs of patients with other diseases might vary. Lastly, this patient navigator was located in a suburb of Chicago in a community-based setting, so extrapolating these findings to other regions and settings needs to be done with caution. While our pilot study used rigorous data collection and analysis methods in this initial examination of community patient navigator activities, additional studies with geographic and demographic diversity that utilize the same protocol are recommended to validate our results.

## 5. Conclusion

Our time in motion analysis suggests that a community patient navigator without research responsibilities is likely to spend more time accompanying patients to appointments relative to completing paperwork and logbooks than navigators engaged in research. While additional time for other activities was expected with the elimination of research responsibilities, we did not expect the time accompanying patients to appointments to demonstrate such an increase relative to documentation. Community navigation may yield greater benefits to patients' clinical experiences as navigators may find more time in their schedules for interacting directly with patients, engaged as a member of the patient's medical care team. By illuminating how patient navigation functions as separate from the research setting, these results may inform future hiring and training of community patient navigators, system design, and operations for improving the efficiency and efficacy of navigators.
